# Children’s Activity Classification for Domestic Risk Scenarios Using Environmental Sound and a Bayesian Network

**DOI:** 10.3390/healthcare9070884

**Published:** 2021-07-13

**Authors:** Antonio García-Domínguez, Carlos E. Galván-Tejada, Ramón F. Brena, Antonio A. Aguileta, Jorge I. Galván-Tejada, Hamurabi Gamboa-Rosales, José M. Celaya-Padilla, Huizilopoztli Luna-García

**Affiliations:** 1 Unidad Académica de Ingeniería Eléctrica, Universidad Autónoma de Zacatecas, Jardín Juárez 147, Centro 98000, Zacatecas, Mexico; antonio.garcia@uaz.edu.mx (A.G.-D.); gatejo@uaz.edu.mx (J.I.G.-T.); hamurabigr@uaz.edu.mx (H.G.-R.); jose.celaya@uaz.edu.mx (J.M.C.-P.); hlugar@uaz.edu.mx (H.L.-G.); 2 Tecnológico de Monterrey, School of Engineering and Sciences, Av. Eugenio Garza Sada 2501 Sur, Monterrey 64849, Nuevo León, Mexico; ramon.brena@tec.mx; 3 Facultad de Matemáticas, Universidad Autónoma de Yucatán, Anillo Periférico Norte, Tablaje Cat. 13615, Colonia Chuburná Hidalgo Inn, Mérida 97110, Yucatan, Mexico; aaguilet@correo.uady.mx

**Keywords:** children’s activity classification, environmental sound, domestic accidents, Bayesian network

## Abstract

Children’s healthcare is a relevant issue, especially the prevention of domestic accidents, since it has even been defined as a global health problem. Children’s activity classification generally uses sensors embedded in children’s clothing, which can lead to erroneous measurements for possible damage or mishandling. Having a non-invasive data source for a children’s activity classification model provides reliability to the monitoring system where it is applied. This work proposes the use of environmental sound as a data source for the generation of children’s activity classification models, implementing feature selection methods and classification techniques based on Bayesian networks, focused on the recognition of potentially triggering activities of domestic accidents, applicable in child monitoring systems. Two feature selection techniques were used: the Akaike criterion and genetic algorithms. Likewise, models were generated using three classifiers: naive Bayes, semi-naive Bayes and tree-augmented naive Bayes. The generated models, combining the methods of feature selection and the classifiers used, present accuracy of greater than 97% for most of them, with which we can conclude the efficiency of the proposal of the present work in the recognition of potentially detonating activities of domestic accidents.

## 1. Introduction

Children’s healthcare is a topic of great global relevance, with different variants and approaches depending on the field where it is applied. One of the important issues in this area is the prevention of domestic accidents. Domestic accidents are one of the main causes of death in children; even the WHO defines them as a global health problem since it is estimated that about 830,000 children die each year as a result of domestic accidents [1]. Children’s domestic accidents are not exclusive to underdeveloped countries; according to UNICEF [2], injuries caused by domestic accidents are responsible for more than 40% of the deaths of children between the ages of 1 and 14 in developed nations. Countries such as Sweden, the United Kingdom, Italy and the Netherlands occupy the last places in the table of infant deaths caused by domestic accidents with an average of 5.2 to 6.6 deaths per 100,000 children, while in countries such as the United States, Portugal and Mexico, there is an average of 14.1 to 19.8 deaths per 100,000 children annually [3]. Regardless of the statistics, the death of children due to domestic accidents is a problem that devastates society and families, regardless of the country or socioeconomic status, and is one that requires urgent attention.

Generally, homes are not designed to be completely safe spaces for children, as there are multiple scenarios and risk objects that can cause accidents for children, such as electrical contacts, stoves, stairs, glass objects, and household appliances, among others. The most common domestic accidents for children are falls, burns, poisonings and hits against objects. The home is where children spend most of their time and, depending on their age, they require adult supervision for many of the activities that they normally do. The parents or guardians of the children are in charge of providing spaces at home that are safe for performing their daily activities, having to consider the possible risk scenarios to adapt to them, thus avoiding domestic accidents as much as possible. Usually, in most cases, children’s domestic accidents occur due to the carelessness of the caregivers of children since it is difficult to monitor them at all times while they are at home, especially when parents work outside the home and they leave their children alone or in the care of someone else.

In recent years, it was common for parents to work outside the home and leave their children home alone for a few hours, which considerably increases the risk of accidents, as, even when left in the care of someone else, accidents happen. During the last year, due to the situation generated by the COVID-19 pandemic, the work scenario of many parents has changed with them having to work at home, but the accidents of children at home have not decreased; they have even increased as collateral damage caused by this health contingency since parents, having to comply with their working hours from home, neglect the activities that their children carry out, which generally causes accidents. Accidents such as trauma, poisoning, burns, choking, the presence of foreign objects in the ears and nose, have increased significantly in both frequency and severity during the period of the pandemic, compared to the same period of the previous year [4]. This makes the emergence of systems focused on children’s healthcare increasingly necessary, especially those designed for the monitoring and prevention of children’s domestic accidents.

Therefore, children’s healthcare has become an area with enormous challenges that are being addressed through artificial intelligence, especially with machine learning and the advantages offered by data analysis applied to this discipline. Monitoring applications focused on children’s healthcare, such as accident prevention, are generally based on the use of an activity classification model. It is precisely this model that is in charge of, through a data source defined for the system, collecting information that allows it to infer the activity that is being performed by the child and identify the activity. These activity classification models are the basis for monitoring systems focused on children’s healthcare.

Most children’s activity classification models that use machine learning techniques use a combination of different sensors that are carried on the garments or parts of the body, which makes them susceptible to such problems as damage due to mishandling, and the continuous replacement of batteries, among others, which can cause data measurements errors. If an activity classification model is used with an invasive data source and is applied to a child monitoring system focused on domestic accident prevention, it is possible that the errors generated by the data source could affect the performance and accuracy of the system. One way to solve the problem of using wearable sensors is to apply another approach to child safety monitoring that uses a different data source.

This paper proposes the generation of a children’s activity classification model, using environmental sound that can be applied in child monitoring systems focused on domestic accident prevention. The activities analyzed in this work are crying, playing (manipulating plastic blocks), running and walking. It was determined to work with this set of activities to highlight the advantage of using environmental sound as a data source by being able to classify activities that are not detectable through movement, such as crying and playing. The objective of generating a model for children’s activity classification using environmental sound is the detection of risk scenarios, that is, if the model detects activities, such as crying, and the child is in an environment with objects that obstruct their walk, then an accident prevention system that uses this model may interpret it as the result of a possible fall of the child or another dangerous event that has triggered their crying. Depending on the environment where children are monitored, activities such as walking, running or playing could represent scenarios susceptible to accidents, for example, running in small spaces or where there are glass objects, a child walking when they are just learning to walk and is not under the supervision of parents, or manipulating plastic objects when they represent a risk of suffocation, among others. This is how this work is presented as a proposal aimed at the area of the prevention of children’s domestic accidents, being that the activity classification models generated here are a fundamental part of the operation of these types of systems, helping to achieve adequate levels of safety and reliability.

For the generation of children’s activity classification models using environmental sound focused on the prevention of domestic accidents, initially, features of the data collected from the environmental sound are extracted and refined through a feature selection process. Then, these data are used to feed a classification model generated using a Bayesian network-based classifier. The objective is for the model to classify the environmental sound data in some of the activities considered for the analysis; this can serve for its use in monitoring systems and the prevention of children’s home accidents through an activity recognition approach. The analyzed feature selection methods are the Akaike criterion and genetic algorithms, while the Bayesian structures used for the generation of the classification models are naive Bayes, semi-naive Bayes and tree-augmented naive Bayes.

This paper is organized as follows: Section 2 presents the work related to the area of children’s activity classification and children’s healthcare. Section 3 presents the background of the feature selection methods used: Akaike’s criterion and genetic algorithms, in addition to the Bayesian classifiers implemented: naive Bayes, semi-naive Bayes and tree-augmented naive Bayes. In Section 4, the materials and methods used for this work are presented. In Section 5, the experiments performed and the results obtained are described. Finally, in Section 6, the discussion and conclusions are presented, as well as the points considered for future work.

## 2. Related Work

The continuous advancement of technology in recent years, especially in the areas of artificial intelligence and machine learning techniques, has led to the development of systems focused on children’s healthcare in different areas, such as those focused on the diagnosis of diseases through symptom detection [5,6], activity classification systems to detect patterns that indicate disease [7,8], or complete child monitoring systems [9,10], among many more. Most of the works developed for children’s healthcare use multiple sensors as a data source, which are embedded in clothing or carried directly by children in some part of the body.

Children’s activity classification is a concept on which numerous works have been developed, the main differences between them being the use that is given to the classified activities and the data source used. The data source used in the children’s activity classification models is a very important aspect for the system since other things depend on it, such as the type of data to be collected, the form of data analysis and the tools used for all phases of the process. As mentioned above, for the works in the children’s healthcare area, most of the proposals for children’s activity recognition models also use sensors embedded in children’s clothing, such as accelerometers, temperature sensors, humidity sensors, RFID devices, or devices carried somewhere on the body, such as a smart watch.

In this sense, Boughorbel et al. [11] developed a children’s activity classification model based on multi-sensor data. They used a device with an accelerometer, a pressure sensor, and a gyroscope; the activities analyzed were walking, running, lying down, falling, climbing stairs, standing up, and other. The multi-sensor device was placed in the back pocket of the children’s pants. The objective was to classify activities for a children obesity prevention system, reaching a classification accuracy of up to 97.8%. Westeyn et al. [12] developed a system based on augmented toys to detect children’s interactions with objects and potentially identify children with autism early. Interaction detection was performed using accelerometers, audio inputs, and contact pressure sensors embedded in specific toys chosen for this purpose. Trost et al. [13] developed a child activity recognition system based on data provided by accelerometers placed on the hip and wrist to detect activities, such as lying down, sitting, walking, running, playing basketball and dancing, resulting in accuracy from 64% to 96% depending on the activity analyzed.

Children’s activity classification works has also focused on children’s healthcare, specifically the prevention of domestic accidents. Nam et al. [14] presented a child activity recognition approach, using an accelerometer and a barometric pressure sensor placed on the child’s waist to prevent childhood accidents, such as unintentional injuries at home. In this work, they analyzed activities, such as wiggling, rolling, standing still, standing up, sitting down, walking, toddling, crawling, climbing up, climbing down, and stopping, achieving an accuracy of 98.43%. Jutila et al. [15] presented a work where they developed a prototype of a portable vest to improve the safety and well-being of children in kindergarten and elementary schools, which contains a GPS, temperature sensors and accelerometers. Through this garment and the sensors it contains, they collected information on the general well-being, behavior and activities of children, being able to categorize activities, such as walking, running, sitting, staying still, as well as data on body temperature and the location of the children.

As mentioned before, using children’s activity classification models based on invasive data sources, such as sensors worn on children’s clothes or on some part of the body, can cause different problems, such as errors in the data collected due to physical failures due to the mistreatment of the sensors or the fact of constantly replacing the batteries. It has been shown that it is possible to use environmental sound as data source in human activity recognition models [16], also obtaining good results when applying the idea to children’s activities [17,18,19,20,21]. Using a non-invasive data source, such as environmental sound, has the advantage of not interfering with the activities that children perform, in addition to potentially having a greater range in the activities that can be analyzed by not focusing only on those that are detectable through motion, as in accelerometer-based models.

When working with activity recognition models through environmental sound, an important process prior to model generation is feature extraction, a process by which a numerical interpretation of the sounds is extracted, which are the so-called features. In the works that involve audio analysis, the most commonly used features are the zero-crossing rate, the signal bandwidth, the spectral centroid, the signal energy and the Mel-frequency cepstral coefficients (MFCC) [22,23], which have also been used in works of activity classification using sound [16].

Generally, after performing the feature extraction, a feature selection process is performed in order to eliminate those features that do not provide relevant information for activity recognition, leaving only those features through which it is possible to differentiate the different classes with which the model works. There are different feature selection methods. Some of them are forward selection, backward elimination, Akaike information criterion (AIC) and genetic algorithms [24]. Both AIC and genetic algorithms have been used in children’s activity recognition models through environmental sound, achieving a reduction in the number of features of 20% and 86%, respectively [17,18].

Once the set of features to be used has been defined, a classifier algorithm is required to generate the model. In the works on children’s activity recognition, there are different classifier algorithms that are implemented, such as random forest, k-nearest neighbor, support vector machines, Bayesian networks and artificial neural networks, among others [14,25,26]. Specifically, in the generation of children’s activity classification models using environmental sound, classical classifying algorithms, such as support vector machines, k-nearest neighbors, random forest, extra trees, gradient boosting and artificial neural networks, are used and compared, reaching accuracies between 40% and 99% [18,19,20].

The present work proposes an approach to children’s activity recognition, using environmental sound as a data source, which can serve as the basis for a child healthcare system focused on the prevention of domestic accidents. By using sound as a data source, the limitation of having sensors carried by the individuals who perform the activity is eliminated, and thus, activities that do not depend on motion detection can be recognized, such as crying, for example. In addition, feature selection processes are performed using the Akaike information criteria and genetic algorithms. For the generated classification model, three classifiers based on Bayesian networks are compared: naive Bayes, semi-naive Bayes and tree-augmented naive Bayes.

## 3. Background

This section presents the background theory of the feature selection methods used: the Akaike criterion and genetic algorithms, as well as the Bayesian classifiers implemented: naive Bayes, semi-naive Bayes and tree-augmented naive Bayes.

### 3.1. Akaike Criterion

The Akaike information criterion (AIC) is a widely used statistical model selection tool [27], which has also been applied to feature selection processes, showing good results [18]. This model selection technique is based on the construction of models considering all the combinations that can be made with the parameters of the model. These models are generated by stepwise regression [28], which is a method of fitting prediction models in which the choice of variables is made through an automatic process of addition or subtraction from the complete set of variables. The following strategies are used:

- Forward selection: In this method, the variables are introduced sequentially into the model. The first variable that is introduced is the one with the highest correlation (positive or negative), and then the other variables that have the highest partial correlation and that are not present in the model are entered. This process is performed until there are no more variables that meet the entry criteria [29,30].
- Backward elimination: In this method, all the variables are introduced into the model and then one after the other, they are eliminated. Variables are eliminated based on correlation, starting with the one with the smallest correlation. The process ends when there are no more variables in the model that meet the elimination criteria [29,30].

The AIC is calculated (AIC is a measure of the relative quality of the models) for each of these models. The best model is the one with the lowest AIC [27,31].

In general, AIC is defined as follows:

(1) AIC=2k−2ln(L)

where:

*k* is the number of model parameters.

ln(L) is the likelihood function for the statistical model.

For smaller data sets, the following correction applies:

(2) $$AICc=AIC+\frac{2k^{2}+2k}{n-k-1}$$

where:

*k* is the number of model parameters.

*n* is the size of the data sample.

Burnham and Anderson [32] recommend using AICs when the ratio between the number of samples and the number of parameters is small (< 40). In our work, considering this proportion, AIC is used. An important point to note about this feature selection technique is that, for the analysis to be valid, the models do not necessarily have to be nested. The advantage of the parameters of one model not being a subset of the parameters of another model is that very different models can be mathematically compared.

### 3.2. Genetic Algorithms

Genetic algorithms are one of the most advanced methods for feature selection and correspond to a technique known as evolutionary computation since they are based on natural genetics and biological evolution [33]. In a natural way, the genes of organisms tend to be in constant evolution with the passage of successive generations to generate and optimize better adaptation to the environment; this is precisely the concept of natural evolution on which the concept of genetic algorithm is inspired.

An advantage of the use of genetic algorithms over other feature selection techniques is that, given its nature, it searches for a wide spectrum of combinations and adds randomness to the search process, which avoids the limitation of probabilistic models and their reduction in combinations. In genetic algorithms, the best solution arises from the best of the previous solutions, that is, an evolutionary algorithm that improves selection over time [34,35,36].

For feature selection through genetic algorithms, we used the genetic Galgo algorithm [37] and the random forest classifier algorithm [38], which demonstrated superior accuracy to the algorithms k-nearest neighbor, nearest centroid, artificial neural networks and recursive partition trees in our previous work of feature selection, using genetic algorithms applied to children’s activity classification using environmental sound [17].

Galgo is an object-oriented package developed in the R language that uses a general aptitude function for feature selection. Galgo initially takes feature subsets as a population and calls them chromosomes. Each chromosome is accuracy-based and evaluated in predicting an output or dependent feature. For this evaluation a classification method is included, such as the random forest classifier. The initial population is replaced by a new one that includes features of different chromosomes, which have greater accuracy in prediction. This process is repeated until the desired accuracy is achieved. This progressive improvement of the chromosome population is inspired by the process of natural selection and the principles of selection, mutation and crossover.

In resume, the application of Galgo consists of four main stages [17]:

1. Setting up the analysis: In this stage, the parameters of the algorithm are set, including the input data, the outcome, the statistical model, the desired accuracy, the error estimation scheme, and the classification method, among others.
2. Searching for relevant multivariate models: This stage consists of the process of selection beginning with the random population of chromosomes, based on a classification method, looking for the best local solutions.
3. Refinement and analysis of the local solutions: The chromosomes selected are subjected to a backward selection strategy since, even when these chromosomes present the best accuracy, there could be features included in the model that do not contribute significantly to the fitness value. The objective of this strategy is to derive a chromosome population containing only features that effectively contribute to the classification accuracy.
4. Development of a final statistical model: Finally, a single representative model is obtained based on a forward selection strategy, where, according to a step-wise inclusion, the most frequent genes presented in the chromosomes population are selected.

### 3.3. Bayesian Network Model-Based Classification

In machine learning, classification is a supervised learning concept in which labels are assigned to data, that is, the class to which they belong is identified. In supervised learning, the classes are previously known and the problem lies in finding a function that assigns each of the objects in the data set to one of the classes. There are a large number of classification approaches and algorithms, each one having a different behavior depending on the nature of the problem in which it is applied; therefore, it is not possible to conclude that one is better than another.

Bayesian networks are probabilistic graphical models that perform probability calculations through Bayesian inference [39,40]. Bayesian networks model dependency based on conditions, representing them by a directed acyclic graph (DAG), where each edge corresponds to a conditional dependency and each node corresponds to a variable [39]. Using the represented relationships, it is possible to make inferences about the variables efficiently, that is, Bayesian networks model a phenomenon through a set of variables and the dependency relationships between them. These models are commonly used in various applications, such as diagnosis, prediction and classification, among others. Before, these models were generated through prior knowledge but recently, with the advancement of machine learning techniques, models can learn from data the two things that define the model: the structure and the parameters associated with the model.

Bayesian network classifiers are competitive performance classifiers and can be simply defined as a Bayesian network applied to the classification process. In this sense, a Bayesian network classifier is defined as the prediction of the probability P(c,x) of some discrete variable *C* (class) given some features *x*, that is, a Bayesian network classifier assigns an object described by a set of features, $X_{1},X_{2},\ldots ,X_{n}$, to one of the *m* possible classes, $c_{1},c_{2},\ldots ,c_{m}$, such that the probability of the class given the features is maximized [41]:

(3) $$Arg_{C}\left[MaxP\left(C|X_{1},X_{2},\ldots ,X_{n}\right)\right]$$

If the feature set $\left\{X_{1},X_{2},\ldots ,X_{n}\right\}$ is defined simply as *X*, then Equation (3) is defined as follows:

(4) $$Arg_{C}\left[MaxP\left(C,X\right)\right]$$

To calculate the probability of the class, given the attributes, the Bayesian classifier uses the Bayes rule as follows:

(5) $$P\left(C|X_{1},X_{2},\ldots ,X_{n}\right)=\frac{P\left(C\right)P\left(X_{1},X_{2},\ldots ,X_{n}|C\right)}{P(X_{1},X_{2},\ldots ,X_{n})}$$

In addition, knowing that $X=\{X_{1},X_{2},\ldots ,X_{n}\}$, Equation (5) is defined as follows:

(6) $$P\left(C|X\right)=\frac{P(C)P(X|C)}{P(X)}$$

So, the classification problem based on the Equation (5), can be expressed as follows:

(7) $$Arg_{C}\left[Max\left[P\left(C|X\right)=\frac{P(C)P(X|C)}{P(X)}\right]\right]$$

If we are interested in maximizing the probability of the class, the denominator P(X) can be considered as a constant, since it does not vary for the different classes:

(8) $$Arg_{C}\left[Max\left[P\left(C|X\right)=\alpha P\left(C\right)P\left(X|C\right)\right)\right]]$$

In Equation (8), it can be observed that to apply the concept of Bayesian networks to classification problems, it is necessary to know the a priori probability of each class, P(C), and the likelihood, that is, the probability of the features given the class, P(X|C), in order to obtain the posterior probability P(C|A). From the above, it is known that for a Bayesian network-based classifier to learn from a data set, it is necessary to estimate the prior probability and likelihood from the parameters of the classifier, that is, the data. The application of Equation (8) results in a very complex system since the term $P(X_{1},X2,\ldots ,X_{n}|C)$ increases exponentially in size as a function of the number of features, requiring a large amount of memory and also a significantly large number of operations to calculate the probability.

In the present work, three Bayesian networks classifiers were implemented: naive Bayes classifier, semi-naive Bayes classifier and tree-augmented naive Bayes classifier. These are described below.

#### 3.3.1. Naive Bayes Classifier

The naive Bayesian (NB) classifier is the simplest of the Bayesian network classifiers and is a classification technique that predicts the class to which an observation or instance belongs, using the Bayes theorem of conditional probability, assuming that the predictor variables are independent and that they follow a normal distribution [42], that is, given the class, each feature $X_{i}$ is conditionally independent of the others: $P\left(Xi|X_{j},C\right)=P\left(X_{i}|C\right),\;\forall j\neq i$. So, Equation (5) can be written as follows:

(9) $$P\left(C|X_{1},X_{2},\ldots ,X_{n}\right)=\frac{P\left(C\right)P\left(X_{1}|C\right)P\left(X_{2}|C\right)\ldots P\left(X_{n}|C\right)}{P(X)}$$

where P(X) is considered a normalization constant.

**Figure 1:**
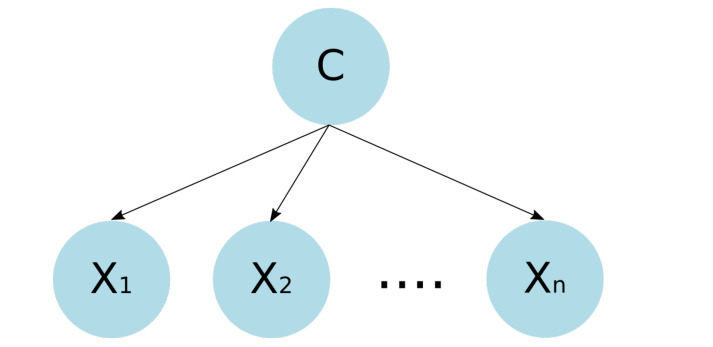
Naive Bayes network structure. Each feature $X_{i}$ is conditionally independent of the others, which can be observed through the distribution of nodes and edges of the DAG.

The implementation of the naive Bayesian classifier considerably reduces the complexity in memory and computation time since the required space increases linearly with the number of features and the number of operations required is also of linear complexity. In Figure 1, the structure of a naive Bayesian network can be observed.

#### 3.3.2. Semi-Naive Bayes Classifier

The semi-naive Bayesian (SNB) classifier tries to maintain the same efficiency as the naive Bayesian classifier but handles the not independent features [43]. For this, two basic operations are considered [44]:

- Deleting a feature. The removal of a feature $X_{i}$ from the classifier can be due to two reasons:
  (a) The feature is not relevant to the class. If the feature does not provide useful information for the classification process, it can be removed.
  (b) The feature is not independent of some other feature $X_{j}$. If two features are highly dependent, they are considered redundant because they provide the same information and one of them can be eliminated.
- Join two features into one. This operation is used when the two features are not independent given the class since, when joining them, this no longer matters.

**Figure 2:**
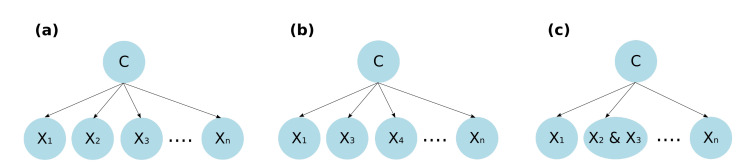
Semi-naive Bayes network structure. (**a**) DAG without operations performed. (**b**) Resulting DAG when performing a feature delete operation. (**c**) Resulting DAG when performing a two-feature into one joint operation.

The decision to implement the operation of elimination or union of non-independent features given the class is based on which of the two approaches causes a greater improvement in the effectiveness of the classifier. Figure 2 shows the representation of the delete and union operations on the DAG that represents the semi-naive Bayesian network.

#### 3.3.3. Tree-Augmented Naive Bayes Classifier

**Figure 3:**
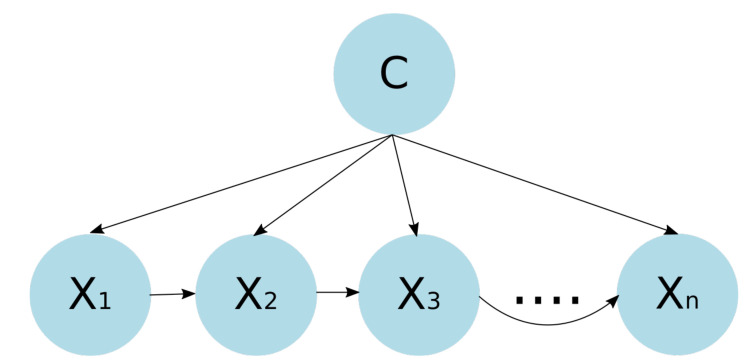
TAN structure. Each feature only depends on the class and one other feature.

The tree-augmented naive Bayesian classifier (TAN) is a semi-naive Bayesian learning method (does not build a complete Bayesian network), which employs a tree structure, where each feature only depends on the class and one other feature [45]. Figure 3 shows the TAN structure. The classifier works by using a weighted maximum spanning tree that maximizes the probability of the data.

Some authors consider that TAN is more consistent and better performing than naive Bayes [46], since in real life, it is common for systems to have related features, and the naive Bayes approach (independent features) rarely happens.

## 4. Materials and Methods

This section describes in detail the data set used for the performed experimentation in this work as well as the followed methodology.

### 4.1. Dataset Description

To perform the environmental sound analysis for the proposed model, the recordings used in our previous work on children’s activity recognition were taken [17,18,19]. The activities considered for this work are those presented in Table 1.

The recordings correspond to children performing the activities alone in a domestic environment, with the aim of resembling as much as possible a child-monitoring scenario focused on the prevention of domestic accidents. The 4 analyzed activities (crying, playing, running and walking) correspond to activities that children commonly perform in domestic environments and that, depending on the place of the house in which they are, may involve dangerous or accident-prone scenarios.

### 4.2. Data Preprocessing

This subsection describes the preprocessing performed on the audio recordings for further analysis. In addition, a pre-analysis performed on the audio samples is described, using principal component analysis (PCA) as an initial method of class grouping.

#### 4.2.1. Dividing Recordings

As was shown in other audio classification works [47,48], the 10 s long recordings contain enough information to be differentiated and classified into the corresponding classes. Many of the used recordings to analyze the activities in the present work have a duration greater than 10 s; that is why, for those that required it, they were divided into *n* 10 s clips. The advantage of dividing the recordings into 10 s clips resulted in an increase in the size of the dataset and the fact of having clips of the same length for later analysis.

#### 4.2.2. Pre-Data Analysis

It is common that, depending on the nature of the problem, statistical methods are used for data analysis, as in the work presented by Vilenchik [49], where data from social media platforms are analyzed using statistics. Considering the above, a pre-data analysis was performed, focused on detecting the feasibility of a feature reduction, using the principal component analysis (PCA) method. In this type of analysis, it is possible to visualize the data groups based on their variance and determine if the reduction can be applied.

### 4.3. Feature Extraction

From the 10 second audio clips, 34 numerical features were extracted, which are shown in Table 2. The feature extraction process was performed using the Python programming language [50] and its pyAudioAnalysis package [51], which contains a wide variety of functions specialized in audio signals analysis.

### 4.4. Feature Selection

The main objective of the feature selection process is to reduce the data set so that those features that do not contribute to the audio classification process are eliminated. The result of the selection process is a feature reduced set that includes only those that are representative of the analyzed activity and that make the audio samples distinguishable and classifiable from each other. There are several methods of feature selection. In this work, we take two of them, which we have previously implemented in the children’s activity classification models: the Akaike criterion and genetic algorithms.

The feature selection processes using the Akaike criteria and the genetic algorithms were performed, using the free programming environment R [52] and the stats [52], mass [53] and galgo [37] packages since R is one of the most widely used statistical languages in the world.

### 4.5. Classification Models

Two different data subsets were used to feed the classification models, one of them obtained through the Akaike information criterion, and the other through genetic algorithms, the two feature selection techniques implemented. For the generation of the classification models, three classifier algorithms based on Bayesian networks were compared: naive Bayes, semi-naive Bayes and tree-augmented naive Bayes. By combining the subsets and the classifier algorithms used, a total of six classification models were generated. The generation of the classification models was performed in the R language.

## 5. Experiments and Results

This section describes the results obtained from the experimentation performed for the generation of the children’s activity classification models, using environmental sound as proposed in this work.

### 5.1. Data Preprocessing

The data set used for this work initially contained 146 audio recordings of 4 different activities performed by children (not the same subject) in a domestic environment independently, that is, one activity at a time and one child at a time. The 4 analyzed activities are crying, playing, running and walking. Table 3 shows the number of recordings available for each of the 4 activities analyzed in this work.

The recordings are of different lengths, and the next step was to divide them into 10-second clips. Table 4 shows the number of clips generated per activity from the original recordings. The total number of 10-second clips, considering the 4 activities analyzed, is 2716.

From the number of recordings per activity shown in Table 3, the division process resulted in a balanced data set in terms of the number of clips per activity, as shown in Table 4. The difference between the activities with the most and fewest audio clips, walking and crying, respectively, is 4.16 % (113 clips).

To perform the analysis of the activities through the generated clips, it is necessary to obtain numerical representations of them, that is, to perform the feature extraction process. The extracted features set is shown in Table 2. When performing the feature extraction process, *NA* values were obtained for some of them in 44 clips, so these clips were not considered for the following processes, reducing the dat set from 2716 to 2672 clips.

**Figure 4:**
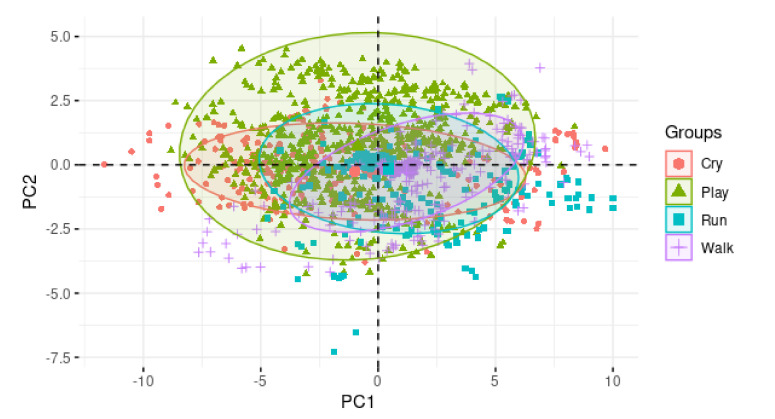
Data clustering based on principal component analysis.

Initially, a data pre-analysis stage was performed in order to verify the feasibility of using statistical methods in reducing features. The principal component analysis (PCA) was used on the complete data set to visualize the grouping of the data, based on their variance and determine the feasibility of applying this method for reduction. Figure 4 shows the groupings generated on a graph, using the first two principal components.

Due to the fact that the PCA-based graph does not show a clear data grouping, based on the variance, the use of combinatorial methods for the feature reduction is preferred and proposed, such as genetic algorithms and Akaike’s information criterion.

### 5.2. Feature Selection

Two feature selection methods were used to compare the performance of the models with each of the subsets generated. The feature selection process caused the data set to shrink, generating different features subsets, depending on the method used. The methods used were the Akaike information criterion (AIC) and genetic algorithms.

#### 5.2.1. Akaike Information Criterion

In the selection process through the AIC, the backward elimination strategy was used for the generation of the models. Thus, we started from an initial model that contains all the features; during the process, those with the lowest correlation were eliminated, generating new models and calculating their AIC. Table 5 shows the AIC calculated for each model generated by eliminating the chosen features following the aforementioned strategy.

The best model is the one with the lowest AIC. Table 6 shows the resulting set of the feature selection process using the Akaike criterion, where the features shown in Table 5 have been eliminated from the original set.

In Table 6 it can be observed that through the Akaike criterion, the number of features decreased from 34 to 27, achieving a reduction in the size of the data set of 20.58%.

#### 5.2.2. Genetic Algorithms

**Figure 5:**
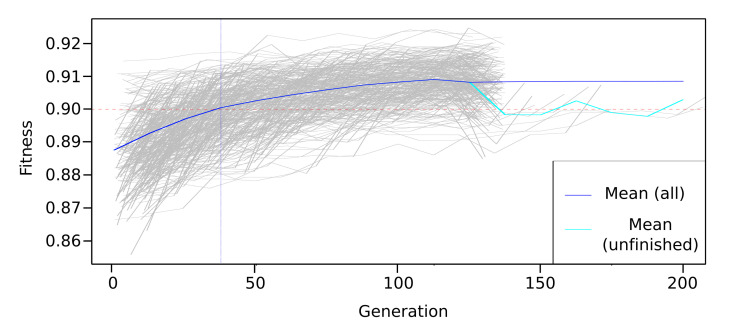
Evolution of the Galgo-generated models for the RF classification method.

From the original data set, the feature selection process was also performed, using genetic algorithms. The Galgo genetic algorithm and the random forest classifier algorithm were used. Figure 5 shows that the performance of the fitness for the generations evolved for the classification method used.

**Figure 6:**
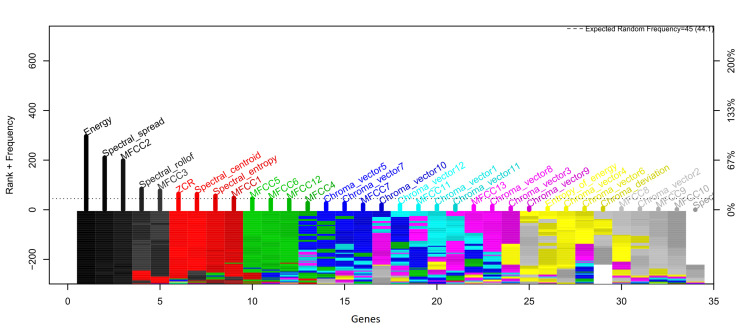
Evolution of the rank with the RF classifier for the most frequent features.

**Figure 7:**
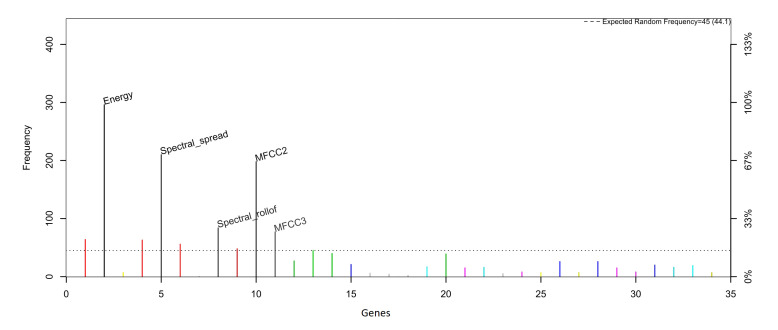
The five features chosen for the RF classifier according to their frequency.

Figure 6 and Figure 7 show the frequency of appearance of the features in the models developed for the classification method used. Those features presented in the color black are the five that presented the highest frequency of appearance and are, therefore, selected as the most representative.

Table 7 shows the resulting features subset from the use of the Galgo genetic algorithm in combination with the random forest classifier algorithm.

By using genetic algorithms as a feature selection method, a significant reduction in the original features set is achieved—from 34 to only 5—which implies a reduction of 85.29%.

The features sets generated in the feature selection process served as a data source for the generation of the Bayesian network models focused on children’s activity classification using environmental sound: naive Bayes, semi-naive Bayes and TAN.

### 5.3. Children’s Activity Classification Models

For the validation of the generated models, a k-fold cross validation approach was used, with k = 10, since it is an approach used in works on human activity recognition to estimate the average accuracy and evaluate the model performance [54,55,56].

#### 5.3.1. Naive Bayes Classifier

**Figure 8:**
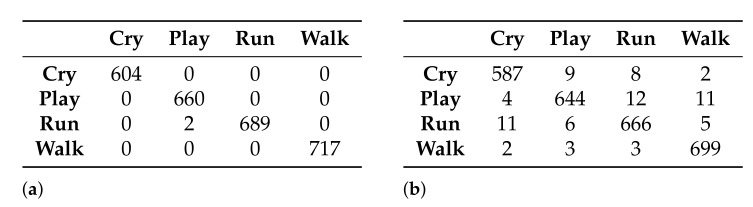
Confusion matrices obtained with the models generated using the naive Bayes classifier. (**a**) Using the 27-feature subset selected by the Akaike criterion and (**b**) using the 5-feature subset selected by the Galgo genetic algorithm.

Two classification models were generated using the naive Bayes classifier: one with the data set generated by the Akaike criterion and the other with the data set generated by the Galgo genetic algorithm. Figure 8 shows the confusion matrices obtained from the generation of the models.

From the confusion matrices of the generated models, the accuracy reached by each model in the classification of the activities can be obtained: 99.92% and 97.15% for the models that use 27 and 5 features, respectively.

**Figure 9:**
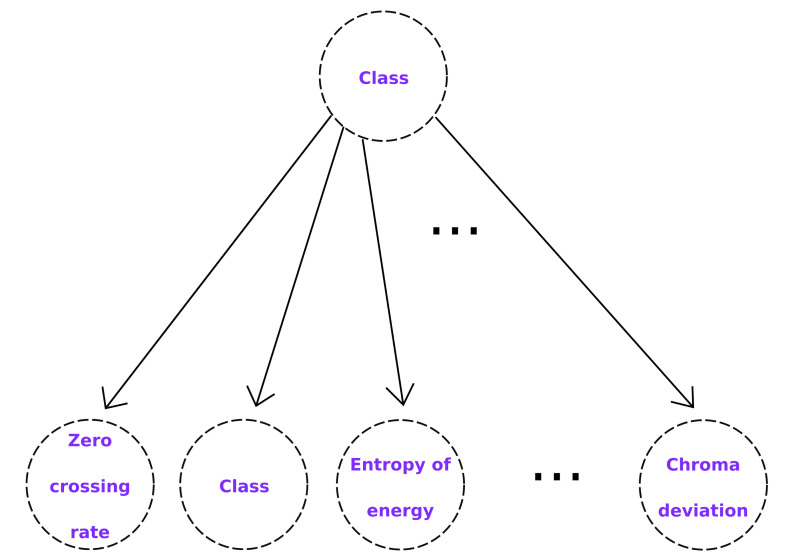
Naive Bayesian network structure generated for the 27-features subset selected, using the Akaike criterion. All 27 features are present and independent of each other.

**Figure 10:**
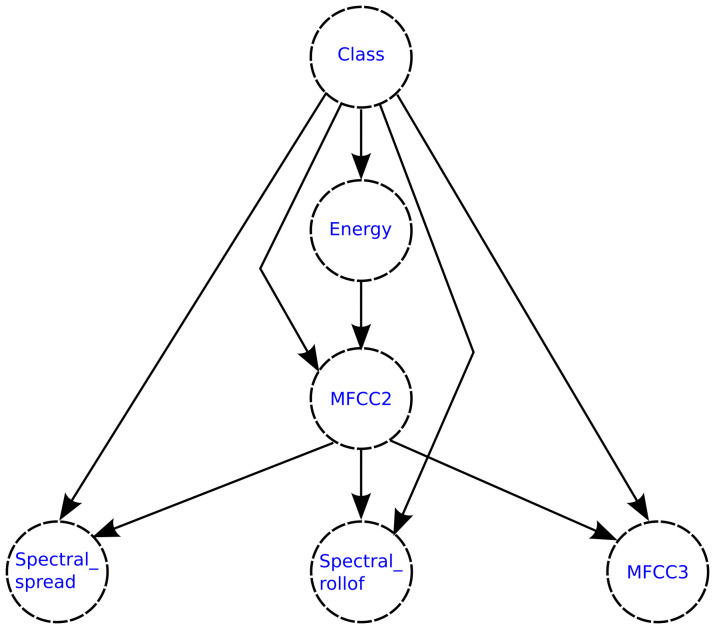
Naive Bayesian network generated for the 5-features subset selected using the Akaike criterion.

Figure 9 and Figure 10 show the structures of the naive Bayes network generated for each classification model, where it is distinguished that each feature is conditionally independent of the others.

#### 5.3.2. Semi-Naive Bayes Classifier

**Figure 11:**
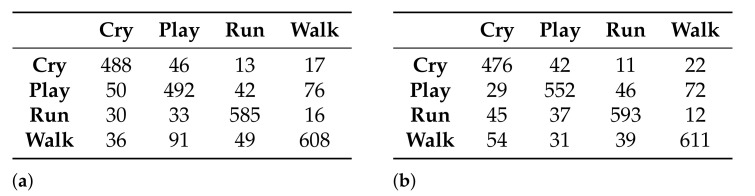
Confusion matrices obtained with the models generated, using the semi-naive Bayes classifier. (**a**) Using the 27-feature subset selected by the Akaike criterion and (**b**) using the 5-feature subset selected by the Galgo genetic algorithm

As was done for the naive Bayes classifier, two classification models were also generated, using the semi-naive Bayes classifier. Figure 11 shows the confusion matrices obtained from each of the models.

From the confusion matrices of the generated models, the accuracy reached by each model in the classification of the activities can be obtained: 81.32% and 83.53% for the models that use 27 and 5 features, respectively.

**Figure 12:**
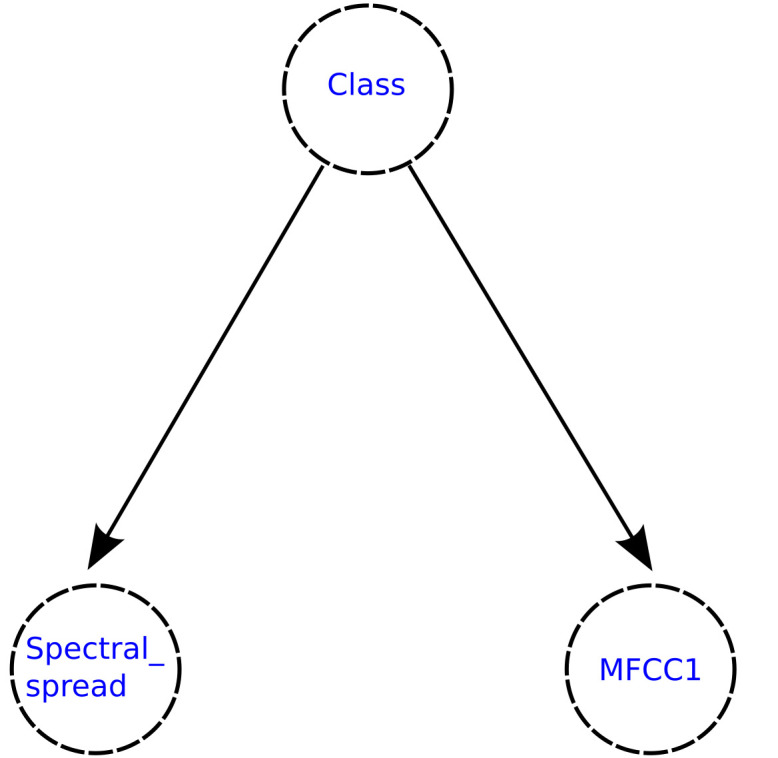
Semi-naive Bayesian network generated for the 27-features subset selected using the Akaike criterion.

**Figure 13:**
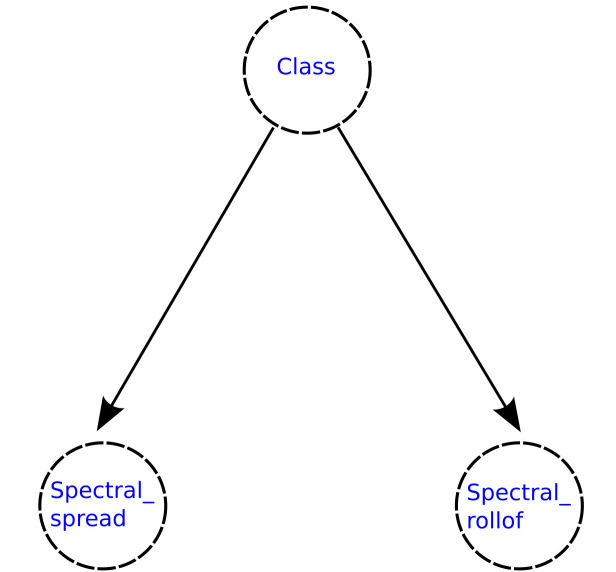
Semi-naive Bayesian network generated for the 5-features subset selected using the Galgo genetic algorithm.

Figure 12 and Figure 13 show the structures of the semi-naive Bayes network generated for each classification model, where it can be observed that the feature delete process was performed for both models, keeping only two features in both cases.

#### 5.3.3. TAN Classifier

**Figure 14:**
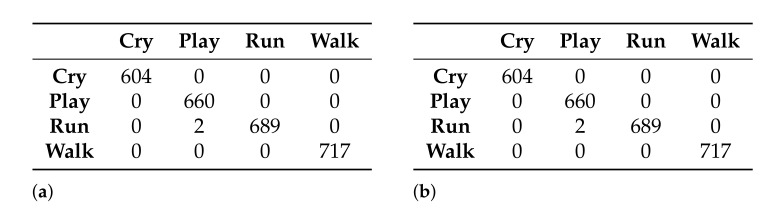
Confusion matrices obtained with the models generated using the TAN classifier. (**a**) Using the 27-feature subset selected by the Akaike criterion and (**b**) using the 5-feature subset selected by the Galgo genetic algorithm

Finally, two models were also generated using the TAN classifier. Figure 14 shows the confusion matrices obtained for the models.

In this case, from the confusion matrices it can be observed that the accuracy for both models is the same—99.92%.

**Figure 15:**
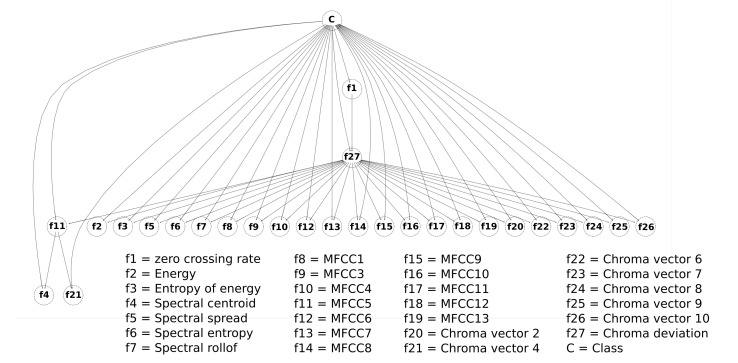
TAN network generated for the 27-features subset selected using the Akaike criterion.

**Figure 16:**
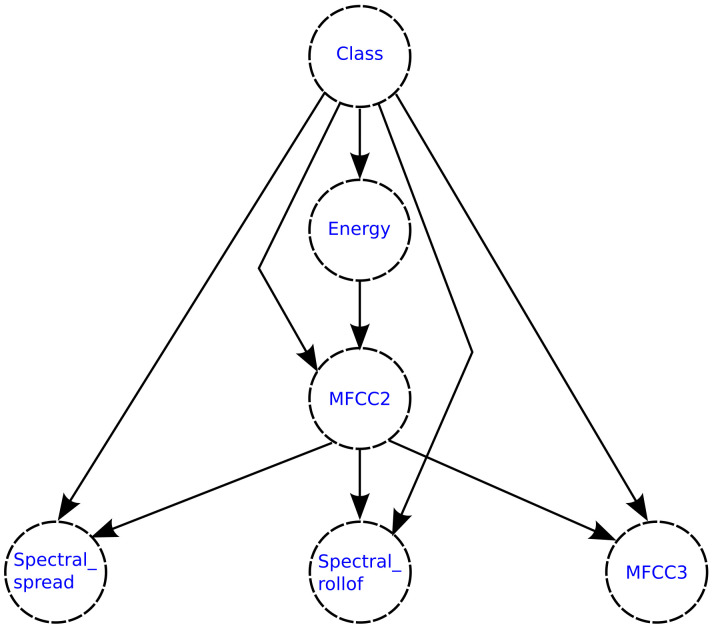
TAN network generated for the 5-features subset selected using the Galgo genetic algorithm.

Figure 15 and Figure 16 show the structures of the TAN network generated for each classification model, where the dependency relationships between the features can be observed.

As summary, Table 8 shows the accuracy achieved by each of the generated models, varying the Bayesian structure and the feature subsets used.

## 6. Discussion and Conclusions

The aim of this work is to generate children’s activity classification models, using environmental sound and applying Bayesian networks-based classifiers as a basis for the identification of potentially dangerous activities in children’s healthcare and monitoring systems. It is also important to have the following considerations:

- The activities analyzed through environmental sound are independent of others. Activities that are performed alongside others require a different analysis.
- In the same way, the data set is made up of recordings of activities performed by children alone, without interaction with others. Activities where there are several children interacting at the same time or where there is external noise require another type of analysis.

For the generation of the classification models, three different Bayesian network structures were compared: naive Bayes, semi-naive Bayes and tree-augmented naive Bayes. In addition, in order to make the models more efficient in terms of resource consumption, two feature selection techniques were implemented, which generated the subsets of data with which the classification models were generated. These techniques are the Akaike criterion and genetic algorithms. From the results presented, the following can be concluded:

- The feature selection methods used reduce the data set considerably, especially the genetic algorithm method, maintaining good levels of accuracy, as in the model generated with the TAN classifier and genetic algorithms as a feature selection method. This model manages to maintain a balance between the accuracy and number of features. The reduction in the data set generally has a favorable impact on the performance of the systems since working with a smaller amount of data generates a lower consumption of resources, an important aspect when the systems are required to be as efficient as possible. However, depending on the application of the classification model, it is preferable to work with a model that maximizes the accuracy of the classification rather than minimizing the amount of data needed for analysis. In an activity classification model focused on being used in a system for the prevention of domestic accidents in children, this aspect is linked to the expected safety for the system. In this sense, it should be noted that two of the models generated using the AIC as a feature selection method achieve an accuracy greater than 99%, while by using genetic algorithms, this is only achieved in one.
- The classification accuracy for the models generated with naive Bayes and TAN is very similar and in both cases greater than 97%, despite the fact that the naive Bayes method is the simplest Bayesian network. As in the previous point, having a method that generates a simple model could favor the performance of the classification system.
- Considering the nature of the problem for which the activity recognition models are to be applied, although all the models generated achieve accuracies greater than 81%, which can be considered good, not all are eligible to be used. It is necessary, as mentioned in previous points, to prioritize maximizing precision over minimizing the amount of data to work with. In this sense, the models that achieve this balance are those generated with the TAN classifier (both feature selection methods) and the model generated with the NB and AIC classifier as the feature selection method.

The results obtained in the children activity classification from the models generated by the Bayesian network approach show similar accuracy to the models generated by other machine learning techniques, such as artificial neural networks and classical classifier algorithms, with the advantage that offers the implementation of a feature selection process, by deleting those that do not provide relevant information for the classification process and generating models that work with only 5 or 27 features without affecting the classification accuracy. In addition, the use of a Bayesian networks-based approach implies the generation of simple classification models, especially with the naive Bayes classifier, which can favorably impact the performance of the system where the classification model is implemented.

Based on the results obtained in this work, it can be concluded that with environmental sound as a data source and by using classifiers algorithms based on Bayesian networks, it is possible to generate children’s activity classification models. These models can be the basis for monitoring systems focused on the prevention of domestic accidents. The activities analyzed in this work (crying, playing, running and walking) are activities that can potentially trigger risk scenarios.

Some points considered for the future are as follows:

- Incorporate more activities analyzed by the classification models to expand possible risk scenarios in child monitoring systems.
- Incorporate the Markovian processes theory for the analysis of activity sequences that allow the identification of complex risk scenarios (cause–effect analysis based on detected activities).
- Generate children’s activity classification models that consider the interaction of two or more children at the same time, as well as simultaneous activities performed by the same child.
- Propose a complete monitoring system focused on children’s healthcare where the classification models generated are used to determine potentially dangerous scenarios based on the activities detected and in combination with other elements, such as the identification of known places.

## Figures and Tables

**Table 1:** General description of activities.

Activity	Description
Crying	Emitting crying sound in reaction to some event
Playing	Handling plastic pieces
Running	Moving quickly from one place to another
Walking	Moving from one place to another at medium speed

**Table 2:** Extracted features.

Feature ID	Feature Name
1	Zero Crossing Rate
2	Energy
3	Entropy of Energy
4	Spectral Centroid
5	Spectral Spread
6	Spectral Entropy
7	Spectral Flux
8	Spectral Rollof
9–21	MFCCs
22–33	Chroma Vector
34	Chroma Deviation

**Table 3:** Number of recordings per activity.

Activity	Recordings
Crying	41
Playing	26
Running	39
Walking	40

**Table 4:** The 10-second clips generated per activity.

Activity	10-Second Clips	Portion of Total Data Set (%)
Crying	604	22.23 %
Playing	703	25.88 %
Running	692	25.47 %
Walking	717	26.39 %

**Table 5:** AIC values for backward elimination process for resulting feature subsets.

Removed Feature	AIC of the Resulting Feature Subset
None	−3353.889
Spectral flux	−3353.889
Chroma vector 1	−3355.872
MFCC2	−3357.755
Chroma vector 11	−3359.062
Chroma vector 12	−3359.983
Chroma vector 3	−3360.481
Chroma vector 5	−3360.590

**Table 6:** Features selected using the Akaike criterion.

Features Selected
Zero Crossing Rate
Energy
Entropy of Energy
Spectral Centroid
Spectral Spread
Spectral Entropy
Spectral Rollof
MFCCs (1, 3–13)
Chroma Vector (2, 4, 6–10)
Chroma Deviation

**Table 7:** Features selected using the Galgo genetic algorithm.

Features Selected
Energy
Spectral Spread
Spectral Rollof
MFCC2
MFCC3

**Table 8:** Children’s activity classification accuracy achieved by the generated Bayesian network models and the selected feature subsets.

	Classifier Accuracy		
Features	NB	SNB	TAN
27	99.92%	81.32%	99.92%
5	97.15%	83.53%	99.92%

## Data Availability

https://drive.google.com/drive/folders/1LBqZ40Xg1Wv8rdJtyA1ZqEUOCLjbIkDF?usp=sharing (accessed on 1 July 2021).
